# Charging Guidance of Electric Taxis Based on Adaptive Particle Swarm Optimization

**DOI:** 10.1155/2015/354952

**Published:** 2015-07-05

**Authors:** Liyong Niu, Di Zhang

**Affiliations:** National Active Distribution Network Technology Research Center, Beijing Jiaotong University, Beijing 100044, China

## Abstract

Electric taxis are playing an important role in the application of electric vehicles. The actual operational data of electric taxis in Shenzhen, China, is analyzed, and, in allusion to the unbalanced time availability of the charging station equipment, the electric taxis charging guidance system is proposed basing on the charging station information and vehicle information. An electric taxis charging guidance model is established and guides the charging based on the positions of taxis and charging stations with adaptive mutation particle swarm optimization. The simulation is based on the actual data of Shenzhen charging stations, and the results show that electric taxis can be evenly distributed to the appropriate charging stations according to the charging pile numbers in charging stations after the charging guidance. The even distribution among the charging stations in the area will be achieved and the utilization of charging equipment will be improved, so the proposed charging guidance method is verified to be feasible. The improved utilization of charging equipment can save public charging infrastructure resources greatly.

## 1. Introduction

In the past forty years, global electric vehicle (EV) technology has gained advancement tremendously [1, 2]. In the last five years, commercial operations of pure electric taxis (E-taxis) have successively begun in Chinese cities such as Beijing, Hangzhou, and Shenzhen, and more than 2,500 E-taxis in total are now in operation. From 2011 to June 2014, a total of 830 E-taxis have been put into operation in Shenzhen. There are now 50 charging stations that can provide charging services to E-taxis, and 648 charging machines/piles are in operation. The operators of these charging stations have established a citywide data monitoring system, which monitors and records operation data of all E-taxis and charging devices in real time.

The usage conditions of charging devices at 15 charging stations in Shenzhen have been surveyed, and charging data of these charging stations in a month were analyzed. To compare the usage rates of the charging devices, the time usage rate (TUR) of the charging devices in a charging station can be defined as follows:

(1) $$\begin{array}{c} \eta =\frac{1}{m}\overset{m}{\underset{j=1}{\sum }}\left(\frac{1}{Z}\overset{n}{\underset{i=1}{\sum }}\left(E_{i}-S_{i}\right)\right), \end{array}$$

where *η* = TUR of the charging devices in a charging station; *m* = number of charging devices; *n* = daily charging frequency of the *j*th device; *E*_*i*_ = end time of the *i*th charging in minute, *E*_*i*_ ∈ [0,1440); *S*_*i*_ = starting time of the *i*th charging in minute, *S*_*i*_ ∈ [0,1440); *Z* = total time of a day in minute; that is, *Z* = 1,440 min.

**Figure 1:**
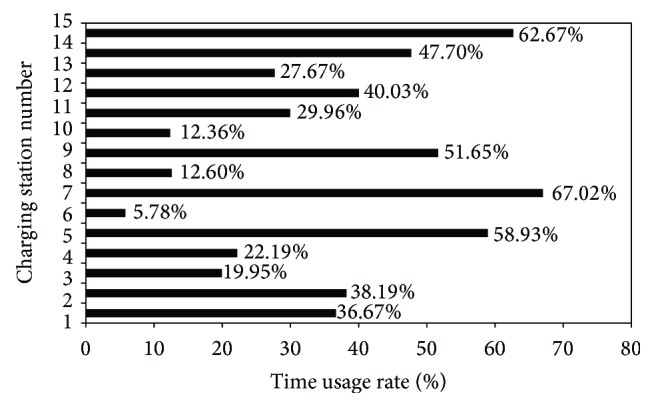
TURs of charging stations in Shenzhen, China.

The TURs of the 15 surveyed charging stations were distributed extremely unevenly (Figure 1). The TURs of several charging stations were relatively high, such that E-taxis had to wait in a queue during a certain period. However, the TURs of other charging stations were relatively low, such that the charging devices were idle for a relatively long time. Therefore, it is necessary to establish an effective system to assist in charging E-taxis more efficiently.

This paper presents the basic framework of a charging guidance system (CGS) for E-taxis, which is based on the data monitoring platform of the charging service network of Shenzhen. And a charging guidance model (CGM) with the adaptive particle swarm optimization (APSO) algorithm is proposed. The correctness of the model and the algorithm is verified through simulation.

## 2. Related Works

In recent years, the multiaspect impacts from connecting a large number of EVs to power grids have become a sustained research focus [2] and coordinated EV charging is the emphasis of the current research [3–10].

In [3], the effect of multiple plug-in hybrid electric vehicles (PHEVs) in a coordinated charging mode on the power distribution network of a residential area was discussed, and a stochastic programming algorithm was proposed. In [4], an admission control mechanism for PHEV charging demand was proposed based on a substation monitoring system. In [5], it was proposed that the EV charging load could be adjusted both temporally and spatially, and it was hypothesized that load scheduling could be performed on a temporal-spatial double scale such that EV charging load could have a positive effect on power grid operation. However, the full load power curve was adjusted only by changing the beginning charging time; spatial charging load scheduling was not realized. In [6], a k-nearest neighbor (KNN) classification algorithm that could predict future charging rates was proposed to select time intervals with low charging rates for PHEVs. In [7], a particle swarm algorithm was used to optimize the charge and discharge control strategy for participating individual EVs in load response. In [8], a multiobjective optimization method was used to improve the mathematical model of a charging scheduling strategy, which solved the problem of poor performance of the charging scheduling strategy in filling the “lowest valley” in a single-objective optimization. In [9], a real-time electricity price-based CGS for EVs was proposed, which could be used to regulate the distribution of EVs at each charging station to improve the voltage quality of the power distribution network. In [10], cost-benefit analysis models for EV discharging users and electric power enterprises were established to calculate the costs and benefits of realizing peak load shifting during peak discharging after the EVs of all parties were integrated into the power grid, which was extremely vital in guiding the EV charging.

The aforementioned studies and other studies have focused on private EVs, their effect on power grids, and coordinated charging guidance strategies. In fact, the effect of public EVs (e.g., buses and taxis) on power grids is more important because these vehicles continuously provide public services and consume more electricity. The charging characteristics of public EVs (E-taxis in particular) are far more complicated than those of private EVs due to their complicated mobility characteristics and a greater number of charging options [11]. There have been few studies that have investigated the charging scheduling of E-taxis. In [11, 12], according to the charging characteristics of PHEV taxis, a backward induction optimization algorithm to select charging time slots was proposed based on the time-of-use (TOU) electricity price and additional charging fees. In [13], a scheduling strategy for a taxi fleet, which included charging plans, was proposed based on clients' demands of taxi reservations to reduce the effect of the charging time and battery swapping on the operational time and revenue.

This paper presents a new viewpoint to enhance the usage rates of large-scale charging devices constructed specially for E-taxis within a city range. The presented research work is based on the collected actual operation data and the presented CGS, CGM, and algorithm are based on the quasi real-time operation data, which makes the presented work different from former research work, having much more practical value.

## 3. CGS Framework

**Figure 2:**
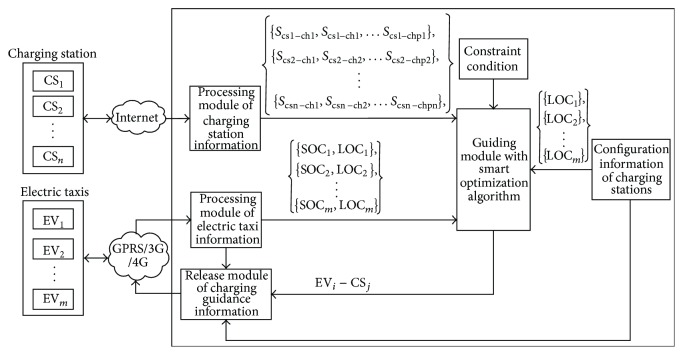
Framework of CGS.

Figure 2 shows the CGS framework for E-taxis charging. By collecting processed information regarding the charging stations (e.g., number and locations of the remaining charging piles) and information regarding E-taxis (e.g., residual capacities of battery packs (SOC) and locations), the control system helps drivers make charging decisions using a smart optimization algorithm when the constraint conditions are met, that is, guides E-taxis to appropriate charging stations.

The CGS sends charging guidance information to each E-taxi in real time at a certain time interval, and E-taxi drivers decide whether or not to respond to such information based on the actual situation. In this way, E-taxis can receive optimized charging guidance when charging is required.

## 4. Charging Guidance Optimization Model

### 4.1. Model Assumptions

When E-taxi drivers select a charging station, they primarily consider factors such as time (including travel time to the charging station, waiting time at the station, and charging time), travel distance, traffic conditions, and charging fees. However, it is expected that the number of E-taxis at each charging station is correlated with the capacity of the charging station to increase the comprehensive usage rate of charging facilities and avoid queuing and resource waste, such as idling charging devices. So, E-taxis' charging is guided optimally as described below.

Under the premise that the calculation of the objective function will not be affected, the following assumptions are made.

1. Within a given region, the number of charging stations (*N*) and the number of E-taxis (*M*) are known.
2. Within an infinitesimal amount of time (Δ*t*), E-taxi drivers can only select one charging station. The flag variable for E-taxi charging is set as *x*_*ij*_, and the value of which is either 0 or 1. When *x*_*ij*_ = 0, the *i*th E-taxi is not charged at the *j*th charging station and when *x*_*ij*_ = 1, the *i*th E-taxi is charged at the *j*th charging station.
3. The impact of traffic conditions is not considered. E-taxis are driven at a uniform speed (*v*_*i*_).
4. When the number of E-taxis waiting to be charged is greater than the number of charging piles at the charging station, some E-taxis have to wait. Therefore, the time required for the *i*th E-taxi to reach the *j*th charging station and complete charging is *t*_*ij*_, which is the sum of travel time to the *j*th charging station *t*_*j*_^*S*^, waiting time at the *j*th charging station *t*_*j*_^*W*^, and charging time *t*_*j*_^*C*^.
5. Waiting time at a charging station is negative linearly correlated with its scale. As the charging station scale increases, the waiting time decreases. Waiting coefficient *δ*_*j*_ is introduced and set as *δ*_*j*_ = 1 − 0.2*G*_*j*_, where *G*_*j*_ ∈ {1,2, 3,4} stands for the charging station scale.

### 4.2. Objective Functions of the Model

The objective function of the model is established by two primary aspects: E-taxi drivers and charging stations within the region.

#### 4.2.1. Aspect of E-Taxi Drivers

For an E-taxi driver, selecting a charging station depends primarily on three aspects: the shortest time, the shortest travel distance, and the lowest charging fees. At the current stage, charging price is fixed. Therefore, time cost and travel distance cost are the primary factors that are considered.

For the time cost, the time required for the *i*th E-taxi to reach the *j*th charging station and complete charging is defined as *t*_*ij*_:

(2) $$\begin{array}{c} t_{ij}=\delta_{j}\overset{M}{\underset{i=1}{\sum }}x_{ij}+\frac{l_{ij}}{v_{i}}+t_{j}^{C}, \end{array}$$

where *l*_*ij*_ represents the distance that the *i*th E-taxi traveled to reach the *j*th charging station in km.

When an E-taxi driver uses charging time *t*_*ij*_ as the basic objective to select a charging station, there is a precedence relationship *ω*_*ij*_ between a specific *t*_*ij*_ value and the minimum value *t*_*ij*_^min^ in set *T*, which includes all the possible charging times:

(3) $$\begin{array}{c} \omega_{ij}=\frac{t_{ij}}{t_{ij}^{min}}. \end{array}$$

The smaller the value of *ω*_*ij*_, the greater the possibility for a driver to select this charging station.

For travel distance cost, the distance that an E-taxi travels to reach charging station *l*_*ij*_ is used as the basic objective to select a charging station. There is a precedence relationship *σ*_*ij*_ between a specific *l*_*ij*_ value and the minimum value *l*_*ij*_^min^ in set *L*, which includes all the possible distances:

(4) $$\begin{array}{c} \sigma_{ij}=\frac{l_{ij}}{l_{ij}^{min}}. \end{array}$$

The smaller the value of *σ*_*ij*_, the greater the possibility for a driver to select this charging station.

The objective function is established from the aforementioned two aspects:

(5) $$\begin{array}{c} F_{1}=min\overset{N}{\underset{j=1}{\sum }}\;\overset{M}{\underset{i=1}{\sum }}\left[x_{ij}\left(\omega_{ij}+\sigma_{ij}\right)\right]. \end{array}$$

#### 4.2.2. Aspect of Charging Station Operators

As can be concluded from the analysis of TUR of charging stations, comprehensive efficiencies of the charging devices are not evenly distributed in the overall service network of charging stations. This uneven distribution means that drivers who are waiting to be charged select the same charging station as the other drivers, which not only reduces the TURs of the charging devices but also negatively impacts the overall development of the charging stations. From the perspective of charging station operators, when E-taxis are distributed based on the scale of the charging stations using the TURs of charging facilities as the objective function is the best. More E-taxis are distributed to large-scale charging stations, and fewer E-taxis are distributed to small-scale charging stations. This demand-based distribution method lowers the fluctuation of the charging load of each charging station and balances the usage rates of the charging piles at the charging stations. Its objective function is the following:

(6) $$\begin{array}{c} F_{2}=min\overset{N}{\underset{j=1}{\sum }}{\left(\overset{M}{\underset{i=1}{\sum }}x_{ij}P_{i}-\frac{C_{j}}{\sum_{j=1}^{N}C_{j}}\overset{M}{\underset{i=1}{\sum }}P_{i}\right)}^{2}, \end{array}$$

where *P*_*i*_ represents the charging power of the *i*th E-taxi in kW and *C*_*j*_ represents the number of charging piles at the *j*th charging station.

#### 4.2.3. Normalization of the Objective Functions

Two objective functions are obtained from the analyses of the two different aspects. Therefore, it is necessary to normalize these objective functions and convert the multiobjective optimization problem into a single-objective optimization problem. The linear weighted sum method is used to convert the objective functions.

Due to the different dimensions of these two objectives, a normalized treatment is necessary for each objective function, as shown in the following equation:

(7) $$\begin{array}{c} F=min\left(\lambda_{1}\frac{F_{1}}{F_{1max}}+\lambda_{2}\frac{F_{2}}{F_{2max}}\right), \end{array}$$

where *F*_1max_ and *F*_2max_ represent the original objective function values before adjustment; *λ*_1_ and *λ*_2_ represent the corresponding weight coefficients of objective functions *F*_1_ and *F*_2_, and *λ*_1_ + *λ*_2_ = 1.

### 4.3. Constraint Conditions of the Model

The constraint condition of the method is that the E-taxis that are waiting to be charged within the region can only select one charging station, as described in the following equation:

(8) $$\begin{array}{c} \overset{N}{\underset{j=1}{\sum }}x_{ij}=1. \end{array}$$

To ensure that E-taxis have enough electric power to reach the designated charging station for charging, the maximum distance *l*_*ij*_^max^(*x*_*ij*_ = 1) that the *i*th E-taxi travels to the *j*th charging station should be less than or equal to the maximum distance *l*_*i*_^max^ that the *i*th E-taxi can travel, as described in the following equation:

(9) $$\begin{array}{c} l_{ij}^{max}\leq l_{i}^{max}. \end{array}$$

In (9), *l*_*i*_^max^ = *L*_*d*_(SOC_*i*_ − SOC_lim_), where SOC_im_ is the lowest limitation generally set by the automaker and *L*_*d*_ is the average maximum driving distance after E-taxis are fully charged according to statistical analysis. Generally speaking, the energy consumption of EVs depends mainly on energy consumption factor, which is related to not only vehicle speed but also road type [14] and makes the relationship between SOC and estimated travel distance a complicated function. So, a linear function is used to simplify the estimation. Because the SOC_*i*_ is updated in time, the estimation accuracy is enough for the charging guidance. This paper sets SOC_im_ as 20% and *L*_*d*_ as 300 km, respectively.

### 4.4. Optimization Algorithm for the Model

The charging guidance for an E-taxi is constrained by various conditions, such as the charging power of the charging station, usage conditions of the charging pile, and charging users' needs. Therefore, the problem is a nonlinear optimization problem with large dimensions, multivariables, and complicated constraints that cannot be solved by classical optimization algorithms, such as linear programming [15]. Here, an adaptive particle swarm optimization algorithm [16] is used.

A particle swarm optimization (PSO) algorithm obtains the optimal value of the swarm by simulating the predatory behavior of bird flocks, that is, by simulating the collective cooperation of bird flocks. The algorithm updates the locations and speeds of the particles using the following equation:

(10) $$\begin{array}{c} V_{id}^{k+1}=\omega V_{id}^{k}+c_{1}r_{1}\left(P_{id}^{k}-X_{id}^{k}\right)+c_{2}r_{2}\left(P_{gd}^{k}-X_{id}^{k}\right),\\ X_{id}^{\left(k+1\right)}=X_{id}^{k}+V_{id}^{\left(k+1\right)}, \end{array}$$

where *ω* represents the inertia weight, which ranges between 0.1 and 0.9; *d* = 1,2,…*D* represents the dimensions; *i* = 1,2,…, *n* represents the particles; *k* represents the current iteration number; *V*_*id*_ represents the speed of the particle *i* in the dimension *d*; *c*_1_ and *c*_2_ are nonnegative constants, which are called acceleration factors; and *r*_1_ and *r*_2_ are random numbers between 0 and 1.

Similar to all the other global optimization algorithms (e.g., genetic algorithms), the premature convergence phenomenon is also present in the PSO algorithm [8], particularly in relatively complicated, multipeak searching problems. Regarding the inertia weight *ω*, if the value of *ω* is a random number between 0.5 and 1, experiments have indicated that the accuracy is greater than that of the linear decreasing strategy, and the convergence speed is also faster than that of the linear decreasing strategy [16]. Here, the value of *ω* is as follows:

(11) $$\begin{array}{c} \omega =0.5-0.5\times rand. \end{array}$$

In this paper, learning factors *c*_1_ and *c*_2_ are accelerated using the nonlinear inverse cosine; *c*_1_ is first large and then becomes small, whereas *c*_2_ is first small and then becomes large. The basic idea is that the self-history information of *c*_1_ is used as the primary reference to search the initial particle flight; the focus shifts to the information of the swarm, that is, *c*_2_ at the later stage [16]:

(12) c1=c1e+c1s−c1e·1−arccos⁡−2k/Maxgen+1π,c2=c2e+c2s−c2e·1−arccos−2k/Maxgen+1π,

where *c*_1*s*_ and *c*_2*s*_ represent the initial values of the iteration; *c*_1*e*_ and *c*_2*e*_ represent the terminal values of iteration; and *Maxgen* represents the total number of iterations of the algorithm.

When we define *f*_*i*_ as the fitness of the *i*th particle (the value of the objective function), the mean fitness of the colony can be obtained using the following equation:

(13) $$\begin{array}{c} f_{avg}=\frac{1}{P_{size}}\overset{P_{size}}{\underset{i=1}{\sum }}f_{i}, \end{array}$$

where *P*_size_ represents the total number of particles.

Then, the normalized scaling factor *f* of the particle swarm is determined using the following equation:

(14) $$\begin{array}{c} f=\left\{\begin{array}{cc} max\left\{\left|f_{i}-\left.f_{avg}\right|\right.\right\}, & max\left\{\left|f_{i}-\left.f_{avg}\right|\right.\right\}>1\\ 1, & other. \end{array}\right. \end{array}$$

Furthermore, the variance of the fitness of the entire swarm *σ* ^2^ can be obtained as follows:

(15) $$\begin{array}{c} \sigma^{2}=\overset{sizepop}{\underset{i=1}{\sum }}{\left(\frac{f_{i}-f_{avg}}{f}\right)}^{2}. \end{array}$$

The extreme value of the swarm, *g*_best_, which meets certain mutation conditions, is mutated according to a certain probability *p*_*m*_. The calculation equation of *p*_*m*_ is as follows:

(16) $$\begin{array}{c} p_{m}=\left\{\begin{array}{cc} \mu , & \sigma_{2}<\sigma_{d}^{2},\;\;f\left(g_{best}\right)>f_{d}\\ 0, & other, \end{array}\right. \end{array}$$

where *μ* is an arbitrary random number between 0.1 and 0.3; the value of *σ*_*d*_^2^ is generally far less than the maximum value of *σ* ^2^; and *f*_*d*_ represents the theoretical optimal value. The method of applying random perturbation was used for the mutation operation of *g*_best_. The parameter *g*_best*i*_ is the *i*th dimensional value of *g*_best_; *β* is a random variable within a Gaussian (0, 1) distribution; then, the mutation of *g*_best_ is as follows:

(17) $$\begin{array}{c} g_{besti}=g_{besti}\left(1+0.5\beta \right). \end{array}$$

Based on the aforementioned analyses, the corresponding solution process of the optimization algorithm can be obtained.

1. Initialize the locations and speeds of the particles in the particle swarm and correct the locations of the particles based on the constraint conditions.
2. Calculate the fitness of every particle (the value of the objective function).
3. Set the extreme value of each particle as the current location and set *g*_best_ as the location of the optimal particle in the initial swarm.
4. Update the speeds and corresponding locations of the particles according to (10).
5. Calculate the fitness of each particle in the particle swarm and update and record the optimal location of each particle and the optimal location of the swarm.
6. Calculate the variation of the fitness of the swarm *σ* ^2^ according to (13), (14), and (15).
7. Calculate the mutation probability *p*_*m*_ according to (16).
8. Obtain a random number between 0 and 1. If this number is less than the mutation probability *p*_*m*_, then execute the mutation operation described in (17) and correct the value of *g*_best_.
9. Determine whether or not termination conditions are met. If true, terminate the calculation. If false, return to step (4).

## 5. Practical Example Simulations

### 5.1. Example Settings

**Figure 3:**
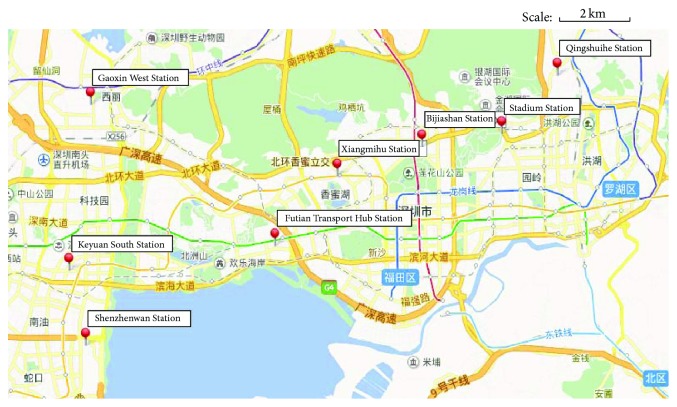
Distribution of example charging stations.

In the present study, eight charging stations and the corresponding mean number of serviced E-taxis in Shenzhen were used as an example; that is, *N* = 8.  Figure 3 shows the distribution of the charging stations.  Table 1 lists the parameters of the charging stations.

The analysis results of the actual operation data show that the initial SOC values of E-taxis when they reached charging stations were normally distributed and the charging time accorded with a 1 h normal distribution. Therefore, the SOC of each E-taxi is randomly selected within a normal distribution *N* (0.5, 0.1) and the charging time of each E-taxi is calculated accordingly. The average charging power is 30 kW; that is, *P*_*i*_ = 30 kW. The number of serviced E-taxis is 100; that is, *M* = 100, and the mean speed on urban roads is 40 km/h; that is, *v*_*i*_ = 40 km/h. The value of the distance that an E-taxi travels to the charging station is randomly selected around the charging station.

### 5.2. Solution Procedure and Simulation Parameters

**Figure 4:**
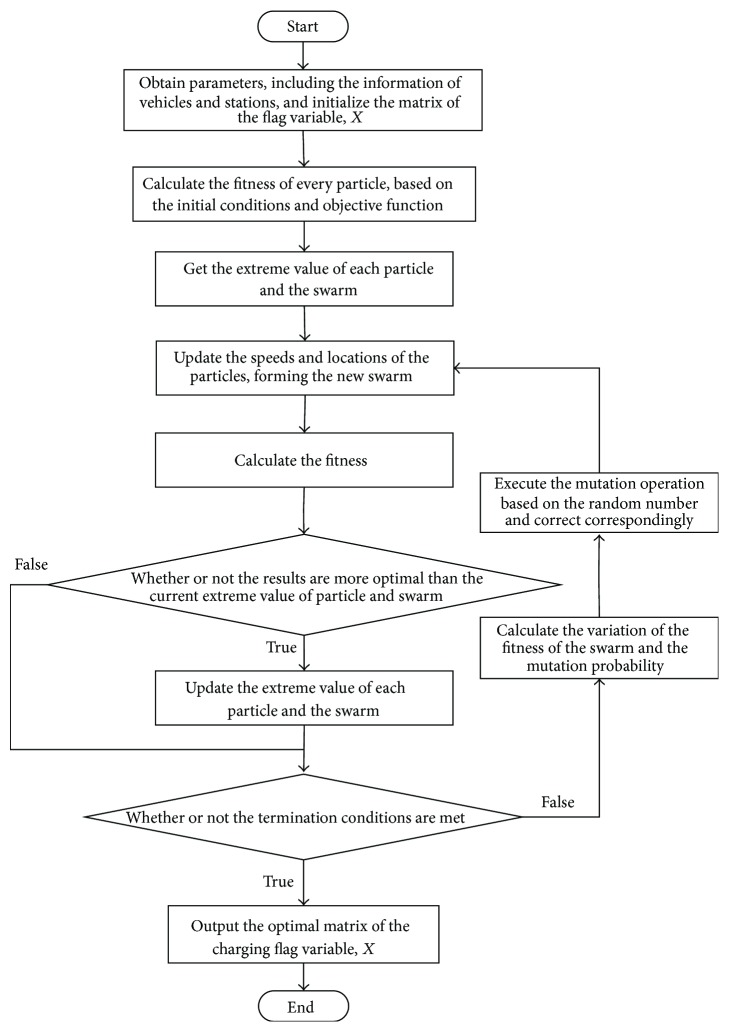
Solution procedure of the example simulation.

According to the charging guidance model and the solution method established in Section 4, the solution procedure of the example is given in Figure 4.

The simulation parameters of the algorithm are as follows: *P*_size_ = 20, *Maxgen* = 300, *c*_1*s*_ = 2.5, *c*_2*s*_ = 0.5, *c*_1*e*_ = 0.5, and *c*_2*e*_ = 2.5, *λ*_1_ = *λ*_2_ = 0.5.

### 5.3. Simulation Results and Discussions

**Figure 5:**
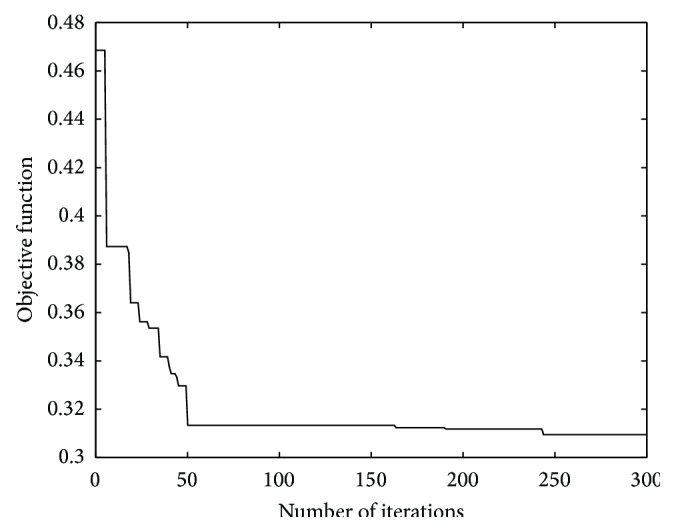
Convergence curve of the objective function.

The simulation outputs the flag variable matrix, *X*, of E-taxis.  Figure 5 shows the convergence curve of the objective function. Based on the value of matrix *X*, the number of E-taxis that reach each charging station can be statistically analyzed as shown in Table 2. In Table 2, because Futian Transport Hub, Shenzhenwan, and Qingshuihe Stations provide service to two types of EVs actually, and the number of charging piles at these stations in the simulation is adjusted, respectively. Table 2 shows that E-taxis can be guided to appropriate charging stations by the CGS, which is based on the number of charging piles at each charging station. The optimal result solves the problem caused by E-taxi drivers' blind selection of charging stations.

**Figure 6:**
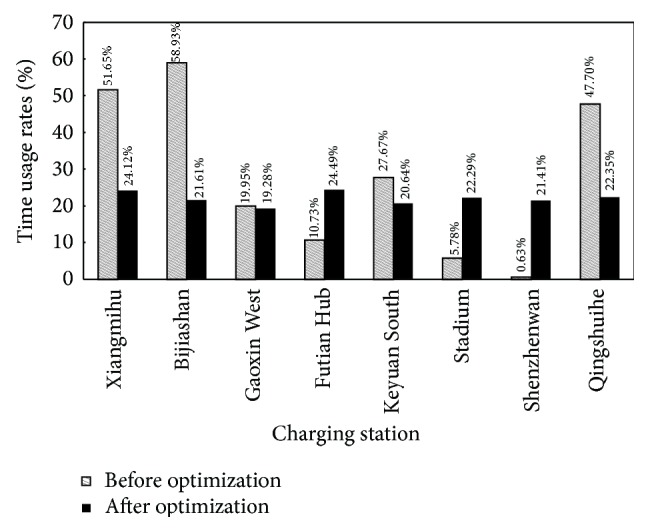
Comparison of the TURs before and after optimization.

In addition, according to the ratio of E-taxis charging time to the total charging time, the TURs of charging devices at Futian Transport Hub, Shenzhenwan, and Qingshuihe Stations are adjusted to 10.73%, 0.63%, and 39.98%, respectively. The comparison between the TURs before and after optimization is shown in Figure 6, which shows that an even distribution of TURs of charging devices among charging stations can be obtained by using the charging guidance method proposed in the present study.

**Figure 7:**
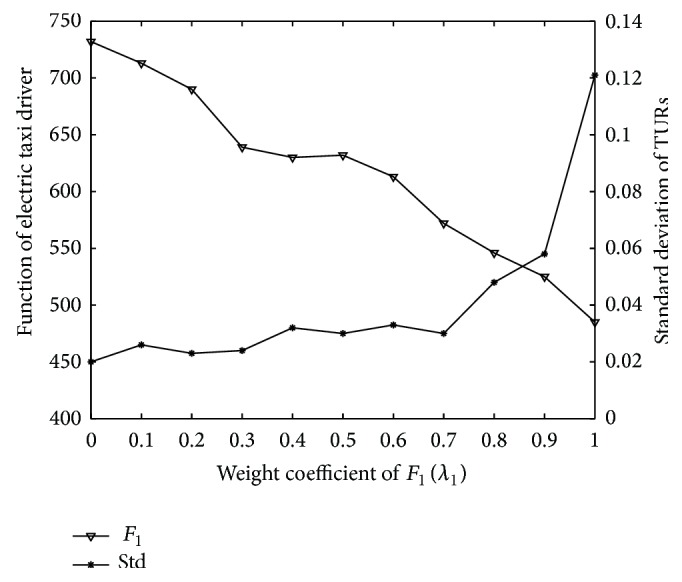
Curves of the results of objective function 1 and standard deviation of TURs.

However, E-taxis driver's benefits would be sometimes sacrificed for even distribution of TURs of charging stations. Different scenarios with varied weight coefficients are simulated to analyze the drivers' loss extent and the standard deviation of the TURs is compared correspondingly. The curve of the objective function 1 and standard deviation of TURs along with the change of weight coefficient are shown in Figure 7.

**Figure 8:**
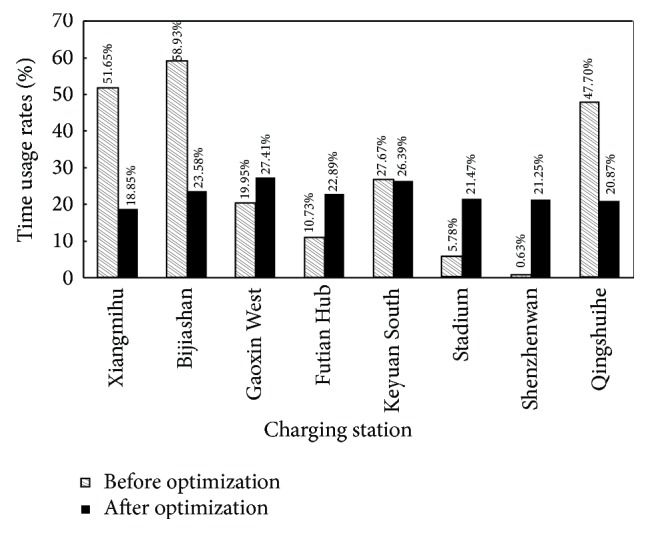
Comparison of the TURs before and after optimization with *λ*_1_ = 0.7.

Figure 7 shows that the function value of E-taxi drivers gradually reduces with the increase of *λ*_1_, while the standard deviation of TURs increases conversely. That means E-taxi drivers benefits could be really sacrificed in order to obtain an even distribution of TURs of charging devices among charging stations. However, the standard deviation of TURs falls much more slowly and tends to be stable when *λ*_1_ is less than 0.7, so weight coefficients can be set as *λ*_1_ = 0.7 and *λ*_2_ = 0.3 to restrict E-taxi drivers' benefits loss less than 18%. The histogram of the comparison between the TURs before and after optimization with *λ*_1_ = 0.7 is shown in Figure 8.

Although the even distribution of TURs of charging devices among charging stations can further sacrifice for E-taxi drivers' benefits, there will be a significant increase in the comprehensive costs of the charging service network after the E-taxis increase and both benefits will be injured. So, the benefit balance of E-taxi drivers and charging station operators can be assured in the specific range.

## 6. Conclusions

This paper proposes a method to calculate TURs of charging devices for E-taxis and conducts a statistical analysis that reveals an extremely uneven distribution of TURs among 15 charging stations in Shenzhen. Therefore, a CGS for E-taxis is proposed, which is based on the information regarding charging stations and E-taxis to solve the uneven distribution problem and improve the comprehensive benefit of charging stations. An optimization model to guide E-taxis charging is established and an APSO algorithm is used to solve the problem. Some simulations are performed based on the actual data of eight charging stations in Shenzhen.

The simulation results show that a more even distribution of TURs of charging devices among charging stations is attained by guiding E-taxis to appropriate charging stations, which verified the feasibility of the charging guidance method proposed in the present study. The benefits of E-taxi drivers and charging station operators can be balanced in the specific range. So, along with the large-scale development of E-taxis, the advantages of the proposed model will become more apparent, particularly regarding public infrastructure resource conservation.

## Figures and Tables

**Table 1:** Parameters of example charging stations.

Serial number	1	2	3	4	5	6	7	8
Charging stations	Xiangmihu Station	Bijiashan Station	Gaoxin West Station	Futian Transport Hub Station	Keyuan South Station	Stadium Station	Shenzhenwan Station	Qingshuihe Station
Number of charging piles	6	8	4	10	4	8	10	6
Waiting coefficient	0.6	0.4	0.8	0.2	0.8	0.4	0.2	0.6

**Table 2:** Comparison of simulation results and actual data.

Serial number	1	2	3	4	5	6	7	8
Charging stations	Xiangmihu Station	Bijiashan Station	Gaoxin West Station	Futian Transport Hub Station	Keyuan South Station	Stadium Station	Shenzhenwan Station	Qingshuihe Station
Number of charging piles in the simulation	6	8	4	10	4	8	10	6
Number of E-taxis being charged in the simulation	10	14	6	18	7	14	19	12
Actual number of charging piles	6	8	4	19	4	8	20	8
Actual number of E-taxis being charged	24	30	4	10	9	3	2	18
